# The evolution of bacterial resistance against bacteriophages in the horse chestnut phyllosphere is general across both space and time

**DOI:** 10.1098/rstb.2014.0297

**Published:** 2015-08-19

**Authors:** Britt Koskella, Nicole Parr

**Affiliations:** Department of Biosciences, University of Exeter, Penryn Campus, Cornwall, UK

**Keywords:** arms race dynamics, fluctuating selection, time-shift experiment, local adaptation, phyllosphere, microbiota

## Abstract

Insight to the spatial and temporal scales of coevolution is key to predicting the outcome of host–parasite interactions and spread of disease. For bacteria infecting long-lived hosts, selection to overcome host defences is just one factor shaping the course of evolution; populations will also be competing with other microbial species and will themselves be facing infection by bacteriophage viruses. Here, we examine the temporal and spatial patterns of bacterial adaptation against natural phage populations from within leaves of horse chestnut trees. Using a time-shift experiment with both sympatric and allopatric phages from either contemporary or earlier points in the season, we demonstrate that bacterial resistance is higher against phages from the past, regardless of spatial sympatry or how much earlier in the season phages were collected. Similarly, we show that future bacterial hosts are more resistant to both sympatric and allopatric phages than contemporary bacterial hosts. Together, our results suggest the evolution of relatively general bacterial resistance against phages in nature and are contrasting to previously observed patterns of phage adaptation to bacteria from the same tree hosts over the same time frame, indicating a potential asymmetry in coevolutionary dynamics.

## 1. Introduction

**Figure 1.**
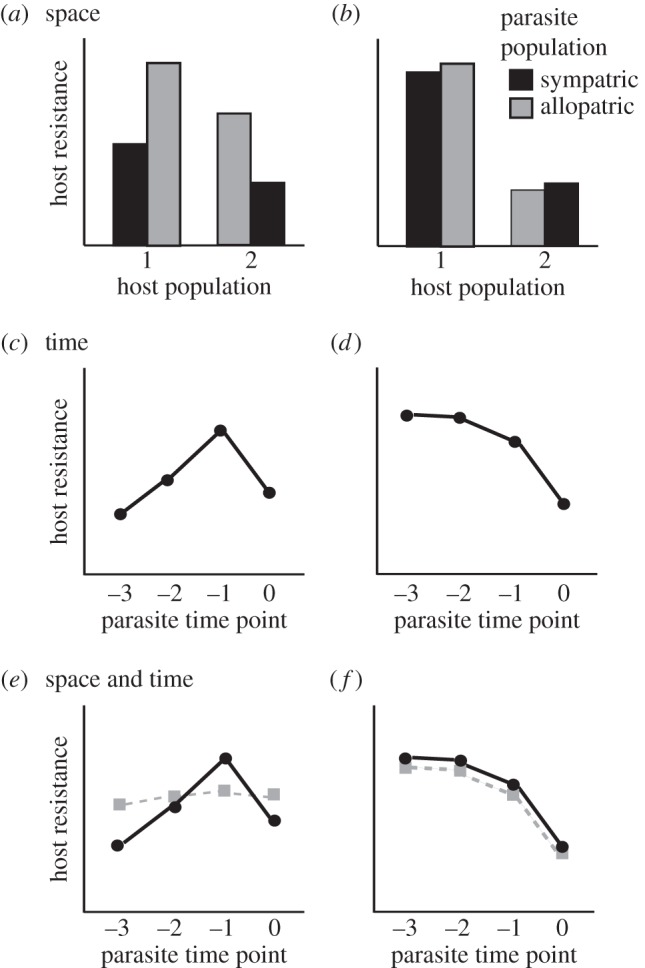
Conceptual illustration of predictions regarding patterns of local adaptation across space and time and the potential to uncover host local adaptation, even when parasites are locally adapted in contemporary time. (*a*) Example of host local maladaptation (and parasite local adaptation) against contemporary parasites from sympatric populations relative to allopatric populations. Such a pattern would be expected when host resistance is specific to local phage-mediated selection. (*b*) Example of no pattern for host local adaptation or maladaptation as would expected when adaptations conferring host resistance are general. (*c*,*d*) Example of time-shift experiment in which hosts are challenged with sympatric parasites from either the contemporary time point (0) or earlier points in time (−1 to −3). The difference between predictions based on a coevolutionary model of fluctuating selection dynamics (FSD; *c*) versus arms race dynamics (ARD; *d*) is illustrated. (*e*) Example of host local adaptation when tested against sympatric (black, solid lines) and allopatric (grey, dashed lines) parasites from the past. In this case, the hosts are maladapted to their contemporary parasites (consistent with (*a*)) and show higher resistance against parasites from the recent past (consistent with (*c*)). (*f*) Example where no host local adaptation or maladaptation is observed, even when tested against parasites from the past. In this case, the hosts are neither adapted nor maladapted to their contemporary parasites (consistent with (*b*)) and show higher resistance against parasites from all past time points (consistent with (*d*)).

The study of coevolution between hosts and their parasites has classically been approached by characterizing variation in infectivity and resistance across space, for example by measuring local adaptation where infection outcome is compared across sympatric interactions (i.e. antagonist populations from the same location) and allopatric interactions (i.e. those from different locations). Local adaptation studies provide evidence for specific adaptations of one antagonist against the other and also offer important insight to the spatial scale at which coevolution is occurring. These data also identify which of the antagonists is better adapted than the other, as local adaptation of one antagonist (i.e. higher fitness when tested against sympatric populations relative to allopatric populations of the other) is typically equivalent to local maladaptation of the other antagonist. The observation from across disease systems that parasites are more likely to be locally adapted to their host populations than vice versa [1,2] is in line with theoretical predictions that parasite populations should have the evolutionary advantage due to their typically higher migration rates and therefore increased additive genetic variation [3,4]. Indeed, manipulation of the rate of migration among experimental populations of bacteria and bacteriophages has been used to demonstrate a positive relationship between parasite dispersal and the magnitude of parasite adaptation to local hosts [5]. A pattern whereby parasite fitness (most commonly measured as infectivity) is higher in sympatric combinations than it is in allopatric combinations suggests both that the parasite/host populations differ across the spatial scale being measured and that parasites have evolved adaptations that are specific to infecting their local host population (figure 1*a*). However, while such results from natural and experimental studies can lend support to the idea that hosts and parasites are following different coevolutionary trajectories across space, they cannot provide direct evidence for coevolution. For example, a pattern of parasite local adaptation could simply reflect one-sided adaptation of parasites to otherwise diverged host populations, rather than implicating parasite-mediated selection as a significant force shaping host populations. Furthermore, a finding of parasite local adaptation does not rule out the possibility that hosts are responding to local parasite-mediated selection. Instead, strong parasite local adaptation could mask any evidence of specific bacterial adaptation.

A more direct way to measure coevolutionary change over time is through the use of ‘time-shift’ experiments, whereby the outcome of infection is compared across hosts and parasites from the same point in time and those that are from either earlier or later in time [6]. For systems in which parasites and hosts can be ‘preserved’ and resurrected, challenging a given parasite/host population against its antagonist from the past, present and future allows measurement of the (co)evolutionary change that has occurred over the time scale being examined (figure 1*c*,*d*). If hosts and parasites are reciprocally adapting to one another, a likely outcome of examining only contemporary combinations across time is finding that overall parasite infectivity and host resistance do not change much, as each new adaptation is offset by a counter-adaptation in the other species. A time-shift approach, on the other hand, holds one antagonist constant in time and examines the fitness of the other across multiple points in time, allowing for adaptation to be tested in the absence of any contemporary or future counter-adaptations. The time-shift approach has most often been applied to microbial systems, as many can survive the freezing and thawing process. In the case of bacterial hosts coevolving with their bacteriophage parasites, this approach has been used to demonstrate ongoing coevolution over the course of experimental evolution (e.g. [7,8]) and has also been used to document coevolutionary change within natural populations [9]. A common outcome of these studies is demonstration that phage populations from the future are more infective to bacterial host populations than phage populations from contemporary or past time points, and reciprocally, that host populations from the future are more resistant to phage populations than those host populations from contemporary or past time points.

Just as local adaptation experiments can offer insight into the spatial scale of adaptation, time-shift experiments can be used to infer the time scale over which adaptation is occurring. In this case, a time shift could be performed over multiple past and/or future time points to uncover coevolutionary dynamics and determine how rapidly adaptations and counter-adaptations are arising and spreading within populations. Moreover, the use of multiple time points can offer important information on the mode of coevolutionary change, for example determining whether coevolutionary dynamics more closely resembles an arms race (ARD) or fluctuating selection (FSD; [6]). Here, a pattern whereby hosts/parasites become directionally more resistant/infective over time (i.e. as the time shift moves further from the contemporary time point) is indicative of ARD (figure 1*d*), as adaptations are generally effective against all antagonists that have not yet counter-adapted, while a pattern whereby hosts/parasites are well adapted to their antagonist from the recent past but are no longer as infective to antagonists from further in the past is indicative of FSD (figure 1*c*). This latter pattern could result from negative frequency-dependent dynamics or trade-offs reflecting costs of resistance/infectivity.

Recent progress has been made in combining measures of adaptation across time and space [10–14] to further characterize the ecological and evolutionary factors shaping a given species interaction or system. In the case of host–parasite interactions, there are at least two novel insights to be gained through these complementary approaches: first, the incorporation of allopatric populations into time-shift experiments can be used to determine whether any observed coevolutionary change is specific to local antagonists, as might be expected under FSD (figure 1*e*), or occurs via a general mechanism, as might be expected under ARD (figure 1*f*; [10]). Second, a combined approach can be used to decouple patterns of host and parasite local adaptation that are normally confounded such that only one antagonist can be locally adapted in contemporary time (parasite local adaptation is equivalent to host maladaptation, but does not necessarily indicate hosts are not responding to local parasite-mediated selection). However, by testing local adaptation against populations of the antagonist from the past, which should not yet have evolved counter-adaptations in response to selection, a signature of local adaptation could be uncovered even when local adaptation is not observed for contemporary combinations (figure 1*e*; [10]). For example, although parasite local adaptation is the more commonly observed finding across systems [1], determining whether or not hosts are specifically adapting to selection imposed by their local parasites could still be measured by testing for host local adaptation against time-shifted parasite populations from either sympatry or allopatry. As parasite populations from the past will not have had the chance to respond to any recently evolved resistance mechanisms, we expect hosts to be most resistant to parasites from the recent past (as is commonly observed in time-shift experiments [7–9]) and can explicitly test whether this increased resistance is specific to local populations; i.e. whether a signature of host local adaptation becomes clear when measured against parasites from the past.

**Figure 2.**
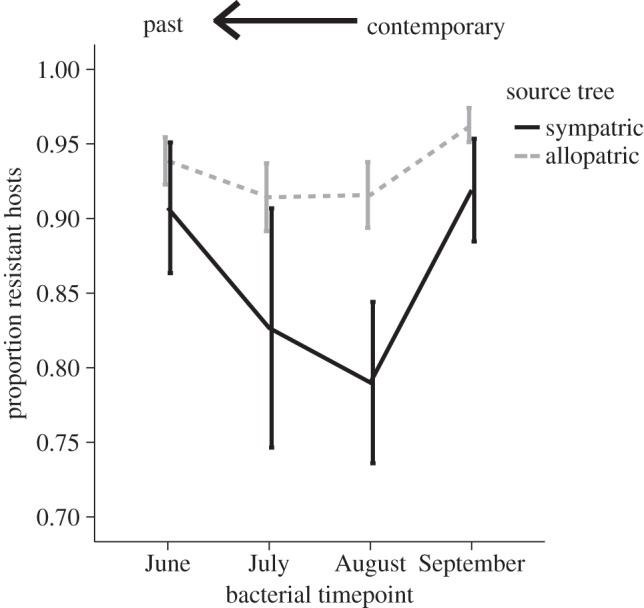
Evidence for phage local adaptation (and bacterial maladaptation) when tested against bacterial communities from the recent past. Solid black line represents sympatric bacteria from the same tree and dashed grey line represents allopatric bacteria from other trees. This figure is adapted from a previous study in which phage populations from September were cross-inoculated against either sympatric or allopatric bacteria from the contemporary time point (Sep) or each of three previous months [10]. However, in this case, host resistance, rather than phage infectivity as in the original figure, is shown on the *Y*-axis to allow comparison with the present data.

Previous work on natural populations and communities of bacteria inhabiting the leaves of horse chestnut trees has demonstrated both high prevalence of bacteriophage viruses (phage) and consistent phage local adaptation at the scale of the host tree, such that phages from a given leaf were able to infect bacterial hosts from either that same leaf or other leaves from the same tree equally well but were generally less infective to bacteria from neighbouring trees [15]. As expected given this clear selection pressure, bacterial hosts were also found to evolve resistance to local phages over the course of the growing season [9]. More recently, this system has been used to combine measures of adaptation across time and space to test the hypothesis that phage local adaptation across space should be more pronounced when measured against hosts from the recent past than when measured against contemporary hosts, which may have already acquired some resistance (figure 2; [10]). Phages were found to be more infective to bacterial hosts from the recent past, and this peak in infectivity (dip in host resistance) was found to be specific to those host bacteria from the same tree rather than being general against all bacterial hosts from the past. Furthermore, phage infectivity was found to be lower again when tested against hosts from four months earlier, a pattern that is more in line with fluctuating selection dynamics [6]. Thus, in line with predictions, the signature of phage local adaptation was found to be higher when measured against time-shifted hosts from the past than in contemporary combinations. In this study, we set out to test the corresponding prediction: that host local adaptation would be observable when measured against time-shifted phage populations (figure 1). By examining bacterial adaptation to sympatric relative to allopatric phage populations from either contemporary or past time points, we explore whether host adaptation is specific or general and whether it is possible to uncover a signature of host local adaptation against time-shifted parasites in a system where parasite local adaptation is the norm [15].

## 2. Material and methods

### (a) Study system

For bacterial populations and communities living in or on eukaryotic hosts, either as pathogens or commensals, there is a clear prediction that the spatial scale of adaptation is likely to be the host individual. Indeed, the composition of bacterial communities has been found to differ markedly both among species [16,17] and among individuals within a species [18–20]. This divergence is likely driven in part by differing patterns of colonization and succession [21], in part by host genetics [20], and in part by within-host species interactions, including competition among bacterial strains/species as well as selection by bacteriophage [22,23]. Lytic phages are obligate killers of their bacterial hosts and can be both highly specific to given bacterial species and found at high prevalence within bacterial populations [24–27]. Phages can impose significant mortality on their bacterial hosts [28,29] and have been shown to select for the rapid evolution of bacterial resistance both in the laboratory and in nature (reviewed in [30,31]). Furthermore, phages can alter the competitive hierarchy of bacterial strains/species [32], facilitate horizontal gene transfer among bacteria [33] and shape the virulence of pathogens [34,35]. Lytic phages recognize specific surface receptors on the bacterial cell and attach to their bacterial hosts using tail fibres, allowing for injection of the phage genome into the host cell [36]. Upon successful infection, the phage hijacks the reproductive machinery of its host cell in order to reproduce new phage particles, and eventually bursts the cell open in order to release progeny into the environment. The ongoing coevolutionary battle between bacteria and phages has resulted in a wide array of host resistance mechanisms [37] and an equally impressive suite of phage mechanisms for overcoming host resistance [38]. However, many of these resistance and infectivity mechanisms are known to carry substantial fitness costs [24,30,32] and therefore are likely to be lost in the absence of selection.

In this study, we focused on communities of culturable bacteria from within leaves of the horse chestnut tree, *Aesculus hippocastanum*, and the lytic phages capable of infecting them*.* Previous work has described the species composition of these bacterial communities from the end of the season [15] and found these to be dominated by bacteria from the Erwinia, Pseudomonas, Pantoea and Rhanella genera, but we do not currently know how this might change over the course of the season. There are reasons to expect these within-host populations and communities to be dynamic over time, even in the absence of phage-mediated selection. Bacterial community assembly within the plant phyllosphere is the product of immigration, survival and growth of competing bacterial species [39], and is therefore subject to a range of environmental and geographical factors leading to seasonal changes in community composition [40]. For example, a study of the leaf-associated microbial communities of cottonwood trees found that bacterial communities were highly variable throughout the season, with temporal variation showing a stronger pattern than between-tree variation on the same day [41]. Furthermore, tree defences are known to be relatively adaptive, especially when compared with short-lived plant species [42], and therefore have the potential to act as a persistent and shifting selection pressure. Similarly, phage populations are likely to show important seasonal dynamics that reflects both the changing density and diversity of their hosts over time, and also the changing abiotic environment [28,43]. This system is ideal for addressing questions of spatial and temporal adaptation, as the spatial scale of interaction can be meaningfully predicted to be the tree host, and the temporal scale of adaptation can be directly examined through freezing and resurrection of bacteria and phages from multiple time points across a growing season.

### (b) Isolation of bacterial colonies and phage inocula from the horse chestnut phyllosphere

In order to test whether a pattern of bacterial local adaptation to phages could be uncovered when examined against time-shifted phages, we crossed bacteria isolated from horse chestnut tree leaves from the end of the growing season against phage populations from either the same tree or neighbouring trees from the contemporary time point and from each of the three previous months. Specifically, to generate starting material for the experiment, two leaves were collected from the same branch of eight different trees, separated by between 25 and 450 m, within a park in Oxfordshire, UK, on four occasions in 2011: 21 June, 23 July, 30 August and 27 September. In order to preserve the microbial and phage communities from the interior of the leaves, samples were brought back to the laboratory and surface sterilized with a 10% bleach, 0.01% Tween detergent solution. They were then rinsed three times with sterile water and placed in a 15-ml Falcon tube containing 0.1 M potassium phosphate (pH 7.2) and 20% glycerol buffer. Tubes were immediately frozen at −20°C until the end of the season, at which point all samples had been collected and individual isolates of bacteria and populations of phages could be extracted.

Frozen samples were prepared by rapid thawing on a shaker placed in a 37°C incubator for 14 min, at which point leaves were homogenized using a Fast-Prep-24 (MP Biomedicals) instrument on a setting of four rotations per second for 60 s with four ceramic beads added to each Falcon tube to aid homogenization. This was found to be sufficient to break up the leaf tissue and to retain the highest bacterial densities. At this stage, a 1 ml sample of each leaf homogenate (approx. 10 ml in total) was preserved at −80°C for eventual isolation of bacterial colonies. The remaining leaf homogenate was then used to generate phage inocula from each sample. To do this, each Falcon tube was centrifuged for 20 min at 550*g* and supernatant was put through a 0.45 µm filter into individual 2 ml aliquots to remove any bacteria present. The leaf homogenates from each pair of leaves from the same tree at each time point were combined when generating phage inoculum in order to produce sufficient phage for the cross. It has been previously shown that there is no significant difference in phage infectivity between leaves when taken from the same tree [15]. The extracted phage samples were then stored at 4°C in the dark.

Frozen aliquots of leaf homogenate from each of two leaves collected in September 2011 from across each of the eight trees were then used to isolate 96 individual culturable bacterial colonies per tree per time point. Specifically, 100 µl of the 1 ml samples of frozen leaf homogenates were diluted in King's broth (KB) to 1 : 10, 1 : 100 and 1 : 1000 strength. A total of 200 µl of each dilution was plated onto a KB 1.2% agar Petri dish and spread evenly across the plate using 5–10 sterile glass beads. Plates were incubated at 28°C overnight. Forty-eight individual colonies per leaf (96 per tree) were then picked using sterile toothpicks and grown individually in 700 µl of KB in a 96 deep well plate. These were incubated at 28°C overnight, at which point 300 µl of 50% KB glycerol buffer was added and mixed by pipetting. Plates were then again frozen at −80°C.

### (c) Cross-inoculation of bacteria and phages across time and space

After all phage inocula and bacterial hosts had been isolated, we tested the susceptibility/resistance of all 768 bacterial isolates from September (96 from each of eight trees) to all 32 phage populations (one from each of eight trees across four points in the season). For this time-shift experiment, one tree (i.e. 96 bacterial isolates) was selected from the freezer at random every week for eight weeks to be crossed against all 32 phages. A 96 pin replicator was used to transfer a small amount of freezer stock into a new 96-well plate containing 200 µl of KB per well, and the 96 bacterial isolates (hosts) were grown up overnight at 28°C. The following day, a standard soft agar overlay was carried out [44]. Briefly, 100 µl of bacteria culture was added to 6 ml of 0.3% KB agar, cooled to approximately 50°C and poured onto a square Petri dish containing a thin layer of 0.6% KB agar. Once cooled, plates were spotted with 10 µl of each of the 32 phage inocula (and two sterile water negative controls) in a grid layout. In addition, each week one negative control (KB only plate) was inoculated with phage to ensure no bacterial contamination within inocula. Plates were incubated upside down overnight at 28°C and then scored for the presence of plaques (a lack of bacterial growth in one section of the lawn caused by phage infectivity) within each spot of inoculum.

To complement the first phage time-shift experiment, where only bacteria from September were examined, we also ran a bacterial time-shift experiment against a fixed phage time point. By crossing bacterial hosts from July against contemporary phages (also from July), we were able to compare this resistance to that of bacterial hosts from September (a future time point) against phages from July. This experiment also allowed us to rule out the possibility that phages were absent or less prevalent earlier in the season (which would give a similar pattern to any observed increases in bacterial resistance against phages from the past resulting from evolved resistance). For this second experiment, we once again isolated 96 bacterial colonies from the freezer stock of non-filtered leaf homogenate from each of eight trees, this time from 23 July 2011. These 768 bacterial hosts were then tested for susceptibility/resistance against phages from the contemporary time point (July) and compared against the susceptibility/resistance of bacteria from September measured in experiment 1 with the same phage inocula. In addition, we measured the resistance of bacteria from July to phages from June in order to measure the signature of local adaptation against this time-shifted combination.

### (d) Statistical analyses

To examine the pattern of host local adaptation across time and space, we first examined variation in bacterial resistance to phages that were either sympatric or allopatric in space and either contemporary or from previous months in time using a generalized linear model with quasi-binomial error distribution, to correct for overdispersion of the data, and the logit-link function. Here, the number of hosts (out of 96) that were resistant to each phage population was treated as the dependent variable and phage time point (i.e. month), sympatry (same or different tree) and source tree were included as factors in the model, along with all interaction terms. The same test was then run to examine the evolution of bacterial resistance against a fixed phage population (from July), but in this case bacterial time point, rather than phage time point, was included in the model.

To directly measure and compare the signature of bacterial local adaptation across time, we used a reciprocal pairwise method that compares differences in the proportion of resistant hosts across sympatric and allopatric combinations [45]. Briefly, a measure of local adaptation was calculated for each pairwise interaction (i.e. bacterial populations from each of the eight trees against phage populations from each of eight trees) as the mean difference (*S* − *A*) between the proportion of resistant hosts to sympatric (*S*) versus allopatric (*A*) phages. For this analysis, an overall mean *S* − *A* measure of zero would indicate no pattern of local adaptation, a negative value would indicate bacterial maladaptation to local phage populations, and a positive value would indicated bacterial local adaptation. A separate analysis was run for each time point, allowing a measure of bacterial local adaptation across space to be calculated and compared across phage time points. To do the latter, a one-way analysis of variance was run comparing mean *S* − *A* values across the four time points. All statistical analyses were run either in IBM SPSS, v. 22 or R v. 3.2 (http://www.R-project.org).

## 3. Results

Of the 737 (out of 768) bacterial hosts from September that were successfully resurrected and tested, 242 (33%) were found to be susceptible to at least one of the 32 phage populations examined (from across both time and space). On average, this subset of hosts was susceptible to 2.08 (1.78 s.d.) of the 32 phage populations. The mean host range of the 32 phage populations was 15.75 host isolates (34.75 s.d.), with eight populations not showing infectivity on even a single bacterial host. For those eight phage populations from September (the contemporary time point), the mean host range was 44.88 host isolates (60.61 s.d.), with all phages able to infect at least one host isolate. Of the 603 (out of 768) bacterial hosts from July that were successfully tested, 285 (47%) were found to be susceptible to at least one of the 16 phage populations examined (from across both time and space). On average, this subset of hosts was susceptible to 2.52 (1.96 s.d.) of the 16 phage populations. The mean host range of the 16 phage populations tested was 44.63 host isolates (44.81 s.d.), with only one population not showing infectivity against even a single host. Interestingly, the mean proportion of resistant hosts when tested against sympatric phage populations from the contemporary time points did not differ between July host populations (0.90, 0.14 s.d.) and September host populations (0.89, 0.25 s.d.), suggesting that bacteria are not becoming more resistant overall through time.

**Figure 3.**
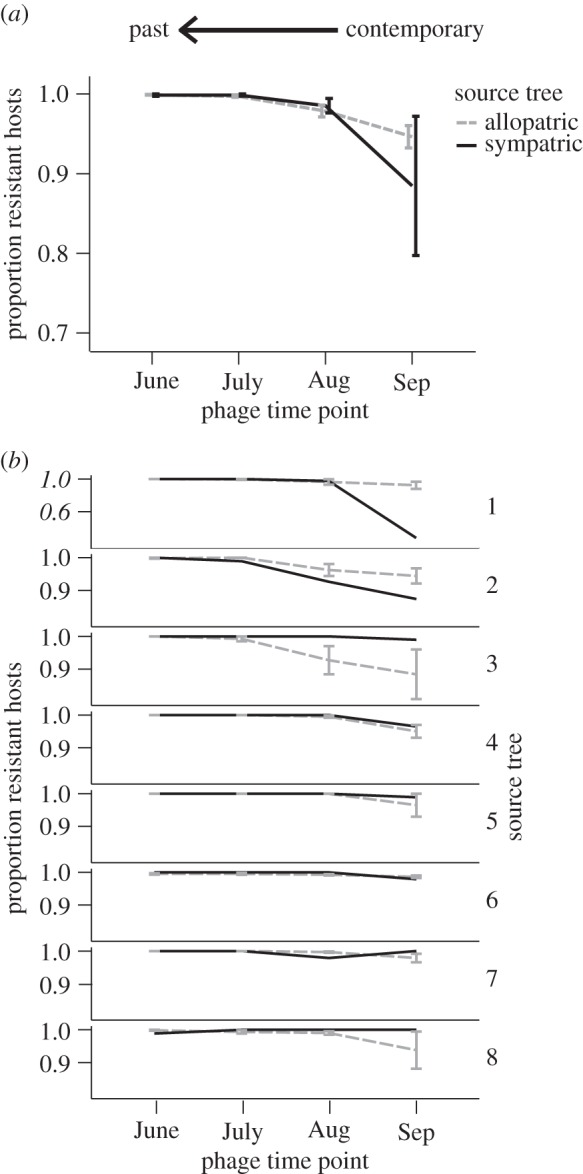
Comparison of the proportion of bacterial hosts from September that are resistant to sympatric versus allopatric phage populations from either the contemporary time point (Sep), a month earlier (Aug), two months earlier (July) or three months earlier (June). (*a*) Shows mean resistance across bacterial communities from all eight trees, while (*b*) shows each bacterial community individually. Note the different scale of the *y*-axis for tree 1. Error bars represent ±1 s.e.m.

The observed pattern of host resistance across space and time indicates that hosts from September are more resistant to phage populations from the past, regardless of whether the phage population is sympatric or allopatric (figure 3). We found significant main effects of source tree (*F*_7,248_ = 6.27, *p* < 0.001) and phage time (*F*_3,245_ = 36.57, *p* < 0.001), as well as a significant interaction between phage sympatry and source tree (*F*_7,216_ = 3.75, *p* < 0.001). There was no main effect observed for phage sympatry (*F*_1,244_ = 3.13, *p* = 0.078), and no interaction effect between source tree and phage time (*F*_21,223_ = 0.66, *p* = 0.868) or phage time and phage sympatry (*F*_3,213_ = 1.29, *p* = 0.278). There was also no observed three-way interaction between phage sympatry, time and source tree (*F*_21,192_ = 0.37, *p* = 0.995). Results were qualitatively similar when analysed using a general linear model, with arcsine square-root transformed proportion resistance as the dependent variable and source tree included as a random effect. When we compared the resistance of bacteria from July versus bacteria from September against either sympatric or allopatric phages from July, we found future bacterial communities were more resistant than contemporary ones, regardless of sympatry (figure 3). In this case, we again found significant main effects of source tree (*F*_7,120_ = 3.95, *p* < 0.001) and phage time (*F*_1,119_ = 62.48, *p* < 0.001), as well as a significant interaction between phage sympatry and source tree (*F*_7,104_ = 2.55, *p* = 0.018). There was no main effect observed for phage sympatry (*F*_1,118_ = 0.78, *p* = 0.379), and no interaction effect between source tree and phage time (*F*_7,111_ = 0.53, *p* = 0.811) or phage time and phage sympatry (*F*_3,103_ = 0.36, *p* = 0.551). There was also no observed three-way interaction between phage sympatry, time and source tree (*F*_7,96_ = 0.00, *p* = 1.000). Again, these results were qualitatively similar when analysed using a general linear model, with arcsine square-root transformed proportion resistance as the dependent variable and source tree as a random effect.

Finally, when we examined the magnitude of bacterial local (mal)adaptation across time for bacterial hosts from September using pairwise comparisons among all phage–bacteria combinations [45], we found no evidence for significant bacterial local adaptation or maladaptation within any time point (table 1). However, the measure of bacterial local maladaptation did change significantly across time, such that mean *S* − *A* differed among phage populations from September, August, July and June (one-way ANOVA, *F*_3,111_ = 3.809, *p* = 0.012). For bacterial hosts from July, we again found no evidence for significant local adaptation or maladaptation against contemporary phages (table 1). There was no significant difference between the July bacterial local adaptation against phage populations from June versus July (*t*_52_ = 1.172, *p* = 0.246). Finally, when we used this same approach to compare resistance of September bacterial populations against contemporary compared with past phage populations (in this case only examining sympatric bacteria–phage combinations), we found a signature of bacterial temporal maladaptation such that bacterial resistance was lower against sympatric phages from the contemporary time point than against sympatric phages from earlier time points (mean contemporary − past time points = −0.1095, 95% CI: 0.094).

**Table 1.** Mean measure of bacterial local adaptation calculated as the proportion resistant to sympatric phage minus the proportion resistant to allopatric phage (*S* − *A*) across all pairwise comparisons within each time shift. Contemporary comparisons are shown in italic. CI, confidence interval.

bacterial time point	phage time point	mean *S* − *A*	95% CI	*t*_27_	*p*-value
July	June	−0.054	0.044	−2.373	0.025^a^
*July*	*July*	*−0.019*	*0.036*	−*1.054*	*0.301*
Sep	June	0.000	0.001	−0.665	0.512
Sep	July	0.002	0.003	1.201	0.240
Sep	Aug	0.006	0.015	0.782	0.441
*Sep*	*Sep*	−*0.054*	*0.055*	−*1.930*	*0.064*

^a^Significance at the 0.05 level.

## 4. Discussion

**Figure 4.**
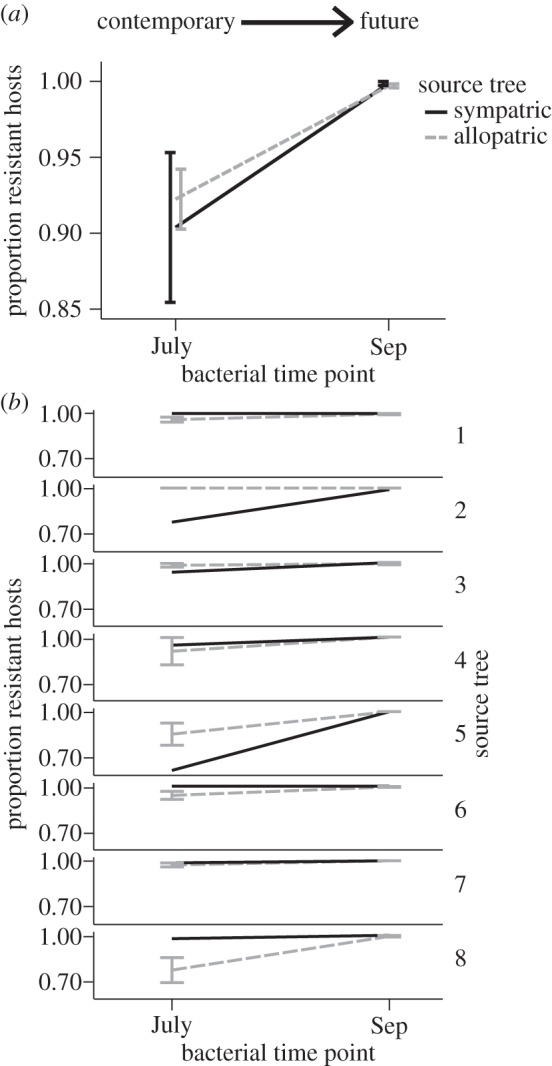
Comparison of the proportion of bacterial hosts from July versus those from September that are resistant to either sympatric or allopatric phage populations from July. (*a*) Shows mean resistance across bacterial communities from all eight trees, while (*b*) shows each bacterial community individually. Error bars represent ±1 s.e.m.

Combining measures of local adaptation across time and space can offer novel and important insight to the dynamics of host and pathogen populations [11,13]. This approach has recently been used to uncover a pattern of peak spatial local adaptation of phages from the horse chestnut phyllosphere against bacterial hosts from the recent past [10]. This previous finding suggests that phage adaptation is both specific to local host populations and short-lived over time, such that phage infectivity is higher against sympatric hosts from the recent past but decreases against phages from earlier in the season (figure 2). In this study, we set out to test the reciprocal prediction: that bacterial local adaptation to phages within the horse chestnut phyllosphere will be more pronounced when tested against sympatric versus allopatric phage populations from the recent past. In line with our predictions and previous work [9], hosts were found to be more resistant to phages from the past (figure 3). However, contrary to our predictions, this increased resistance was found to be general across both space and time, as bacteria were more resistant to both sympatric and allopatric phages from the previous month and showed no evidence of decreasing resistance against phages from earlier in the season. We can rule out the possibility that the observed increased resistance against phage populations from earlier in the season is the result of lower overall prevalence of phages at these time points, as bacterial hosts from July were found to be highly susceptible to these same inocula from both June (data not shown) and July (figure 4). Furthermore, bacteria from September were found to be more resistant to phages from July than were contemporary bacteria from July (figure 4), and again this pattern was consistent for both sympatric and allopatric phage populations. Together, the results suggest that, unlike phage infectivity from the same populations and over the same time series [10], adaptations conferring bacterial resistance are remarkably general and long-lasting over the course of the growing season.

As highlighted in a conceptual review [6] and demonstrated in a number of empirical laboratory studies [46–48], time-shift experiments can be extremely useful in clarifying whether a given coevolutionary interaction is more in line with fluctuating selection or arms race dynamics. The spatial and temporal results from this study are suggestive of ARD, rather than FSD, as bacterial hosts were found to be more resistant to phage populations from all three time points in the past. This result is particularly surprisingly in light of previous results from the corresponding phage populations, which was suggestive of adaptation specific to local host populations and FSD (figure 2; [10]). However, the results are not unlike those commonly observed in the laboratory. For example, *in vitro* coevolution between *Pseudomonas fluorescens* and phage SBW25φ2 lead to a pattern whereby hosts evolved increasing resistance against a wider and wider range of phage types over time [49]. There is, however, one relative limitation in the interpretation of coevolutionary dynamics from time-shift experiments: patterns consistent with ARD could also simply reflect too narrow a window for observation, such that any decrease in resistance/infectivity may have been missed by not going back further enough (or indeed ahead further enough) in time [6]. In this study, the absolute time examined was the same for the bacteria and phage populations (four months), but the relative windows of observation may differ when generation times of each player are taken into account, as phages typically have a shorter generation time than their host. In particular, if the loss of infectivity/resistance against bacteria/phages from the past is driven by fitness costs, phage populations may respond to selection against these costly adaptations more rapidly than their host populations. Alternatively, phage adaptation may be more constrained by their typically smaller genome sizes, such that counter-adaptation to a newly resistant bacterium comes at the cost of previous adaptations. If this were the case, bacterial populations might still show decreased resistance if you went further back in time, for example to a previous season. Even if such dynamics was uncovered, however, there still appears to be a fundamental difference in specificity across the two antagonists, such that while phage infectivity against past hosts is higher against sympatric than allopatric populations, host populations are equally more resistant to sympatric and allopatric phage populations from the past. Furthermore, given the seasonal constraints of life within tree leaves from a temperate region, the observed differences across the season are likely to represent a realistic window of time in which the coevolutionary interaction is taking place before potentially being disrupted or restarted.

Importantly, the increasing resistance of bacterial hosts from September observed across both sympatric and allopatric phage populations from the past does not mean that phage prevalence decreases over the course of the season. The similarity in the proportion of hosts that are resistant to their sympatric, contemporary phage populations in July (figure 4) and September (figure 3) suggests instead that both antagonists are continually evolving in response to one another and that the overall prevalence of infection remains relatively unchanged. Nonetheless, the observed lack of any loss of resistance against phages from the past suggests the evolution of a relatively general and cost-free resistance mechanism. Work from across bacteria–phage systems has demonstrated fitness costs associated with resistance [32,50–52], but these costs are not universally found [53,54] and are likely to vary depending on the environment [55] and the mechanism of resistance used [30]. For example, the bacterial CRISPR-Cas system can confer resistance against an increasing range of phages as new phage ‘spacers’ are introduced into the bacterial genome [56], presumably without the acquisition of additional fitness costs to the host. On the other hand, loss or alteration of surface receptors on the bacterial cell can confer general resistance against many phage types but is likely to be quite costly, especially in the context of the phyllosphere [57]. Differences in the realized costs of resistance across environments have been shown experimentally to alter the mode of coevolution. For example, increasing the supply of nutrients was observed to shift coevolutionary dynamics of experimentally evolved bacteria and phages from FSD towards ARD [47], as bacteria were able to invest in and maintain more costly resistance over time. Similarly, increasing the rate of mixing within populations from semi-natural soil microcosms was again shown to move the coevolutionary dynamic from FSD towards ARD [46], as the rate of encounter (and therefore the strength of selection) was increased under higher mixing. An alternative explanation for the observed increased resistance against past phages, regardless of sympatry, is that it is not general *per se*, but rather is the result of selection imposed by the same population/community of circulating phage types throughout the season as they disperse among trees. In other words, the observed cross-resistance against allopatric phages could represent past local selection by those same, or similar, phages.

The finding that hosts were not locally adapted or maladapted to phages from contemporary time points (in either July or September), and if anything were likely to be slightly maladapted (table 1, although note large differences among trees in figure 3*b*), is in agreement with both previous work demonstrating consistent phage local adaptation across horse chestnut trees [15] and theory suggesting parasites are often ‘ahead’ in the coevolutionary battle [3,4]. Previous studies from across bacteria–phage systems have also demonstrated that phage populations are more likely to be locally adapted to their hosts than vice versa [45,58,59], as might be predicted based on their shorter generation times and potentially greater population sizes. However, here we show that bacterial local adaptation is also not observed against time-shifted phages, suggesting that the pattern of phage local adaptation observed previously is not simply a reflection of phages being better adapted than their bacterial hosts but rather might be indicative of fundamental differences in specificity between phage infectivity and host resistance. Some insight to this asymmetry can be gleaned from experimental evolution of the bacterium *P. fluorescens* SBW25 and its phage Φ2. Although coevolution in microcosms has been shown to lead to directional increases in both the range of host resistance and phage infectivity [7], recent evidence suggests that the host range of a given phage does not influence either the ability of the host to evolve resistance to it or the range of resistance evolved (i.e. the cross-resistance to other genotypes) [60]. The asymmetry observed in both the experimental system and the natural horse chestnut system could reflect differences among mechanisms of resistance and infectivity. In particular, while phages must evolve specific adaptations to recognize and attach to their host cells, bacterial hosts can evolve resistance through the loss or alteration of attachment sites on the cell surface, therefore acquiring cross-resistance against all phages using that receptor which have not specifically counter-adapted to this modification/loss [60]. Of course, the results of this study reflect a pattern from across a diverse community of culturable bacterial isolates from the phyllosphere [15] rather than a specific pairwise coevolutionary interaction. Thus, the results do not rule out specific local adaptation of individual bacterial strains or species to their corresponding phages and may be indicative of changing composition of phage types over the course of the season as the host community composition changes (figures 3 and 4). For instance, phages from June could represent an entirely different subset of types than phages from September, and these subsets could use different mechanisms of infection that are specific to their local, contemporary hosts. In this case, all non-hosts from either earlier or later in the season and from either sympatric or allopatric populations would be resistant.

## 5. Conclusion

Host local adaptation across space has rarely been documented despite a large number of studies from across a wide breadth of systems [1,2]. This lack of observed host local adaptation could simply be considered a consequence of stronger parasite local adaptation masking any patterns of specific host adaptation. Our results suggest that this might not be the case. For interacting bacteria and phages in the horse chestnut phyllosphere, it seems instead as though the specificity across space and durability across time of parasite adaptation may fundamentally differ from that of host adaptation. While phage local adaptation is more often observed than bacterial local adaptation when measured across contemporary combinations [15], and the signature of phage local adaptation becomes more pronounced when tested against bacterial hosts from the recent past [10], we find no evidence for specific bacterial resistance against sympatric phage populations from either contemporary or past time points. Application of this combined local adaptation time-shift approach to other systems will allow the generality of this surprising asymmetry to be tested.

## Data accessibility

Data included in this study are deposited in Dryad (http://dx.doi.org/10.5061/dryad.q0d23).
